# Supplementary material for: Therapeutic mitigation of measles-like immune amnesia and exacerbated disease after prior respiratory virus infections in ferrets
Source: Nat Commun. 2024 Feb 8;15:1189. doi: 10.1038/s41467-024-45418-5 (PMC10853234; doi:10.1038/s41467-024-45418-5)
Supplement: Supplementary file 1 — Supplementary Information [file 41467_2024_45418_MOESM1_ESM.pdf]

Therapeutic mitigation of measles-like immune amnesia and exacerbated disease after prior respiratory virus infections in ferrets

Robert M Cox, Josef D Wolf, Nicole A Lieberman, Carolin M Lieber, Hae-Ji Kang, Zachary M Sticher, Jeong-Joong Yoon, Meghan K Andrews, Mugunthan Govindarajan, Rebecca E Krueger, Elizabeth B. Sobolik, Michael G Natchus, Andrew T Gewirtz, Rik deSwart, Alexander A Kolykhalov, Khan Hekmatyar, Kaori Sakamoto, Alexander L Greninger, Richard K Plemper

Supplementary Information table of contents:

1) Supplementary Table S1. GHP-88309 single oral dose plasma PK in ferrets
2) Supplementary Table S2. GHP-88309 repeat oral dose plasma PK in ferrets
3) Supplementary Table S3. DNA primers used in this study
4) Supplementary Figure S1. Single-dose oral PK study of GHP-88309 in ferrets
5) Supplementary Figure S2. Non-formal tolerability study with GHP-88309
6) Supplementary Figure S3. GHP-88309 repeat-dose plasma PK
7) Supplementary Figure S4. α-CDV and α-RABV nAbs titers in ferrets
8) Supplementary Figure S5. Clinical signs of ferrets infected with recCDV-5804p
9) Supplementary Figure S6. Clinical disease after infection of ferrets with recCDV-5804p NΔ425-479 or recCDV-5804p Nectin 4-blind
10) Supplementary Figure S7. CBC results after infection of ferrets with recCDV-5804p NΔ425-479 or recCDV-5804p Nectin 4-blind
11) Supplementary Figure S8. Clinical presentation of recCDV-5804p NΔ425-479-infected and GHP-88309-treated ferrets
12) Supplementary Figure S9. Selected cytokines profiles after flagellin-stimulation of ferrets recovered from CDV
13) Supplementary Figure S10. IAV challenge of ferrets after recovery from CDV disease
14) Supplementary Figure S11. Challenge of GHP-88309-treated ferrets with CDV-5804p after recovery
15) Supplementary Figure S12. Clinical disease after infection of ferrets with pdmCA09
16) Supplementary Figure S13. α-H1N1 nAbs titers in ferrets before infection with CDV
17) Supplementary Figure S14 Exacerbated lung disease after consecutive infection of ferrets with IAV and

CDV

18) Supplementary Figure S15. Necropsy of consecutively infected ferrets presenting moribund
19) Supplementary Figure S16. All histopathology analyses
20) Supplementary Figure S17. Histopathology scores, Gram stains, and metagenomics after consecutive infection of ferrets
21) Supplementary Figure S18. Effect of treating primary H1N1-pdmCA09 infection prior to CDV
22) Supplementary Figure S19. Exacerbated disease after primary infection with RSV followed by recCDV-5804p NΔ425-479
23) Supplementary Figure S20. Selected cytokines profiles after IAV and CDV infection of ferrets
24) Supplementary Figure S21. Expression of TFFs and Muc5 proteins in singly or consecutively infected ferrets
25) Supplementary Figure S22. Shed pdmCA09 titers after primary infection of ferrets
26) Supplementary Figure S23. CBC results of consecutively infected ferrets treated with GHP-88309
27) Supplementary Figure S24. α-CDV nAbs titers in GHP-88309 experienced or inexperienced ferrets after recovery from CDV

Supplementary Tables

Supplementary Table S1. GHP-88309 single oral dose plasma PK in ferrets. Selected plasma PK parameters of GHP-88309 in ferrets after a single oral dose of 50 or 150 mg/kg.

ID	**dose**[A] [mg/kg]	t_{max} [hours]	C_{max} [nmol/ml]	**AUC-INF** [hours×nmol/ml]	**AUC-INF/dose** [hours×nmol/ml/mmol]	$t_{1/2}$ [hours]
GHP-88309	50	2	55.1 ± 27.5	177.8 ± 71.5	947.3 ± 381	1.5 ± 0.3
GHP-88309	150	3.3 ± 2.3	92.8 ± 30.6	754.1 ± 731.7	4017.5 ± 3898.4	2 ± 0.29

[A]Data analysis with WinNonLin; n=3.

Supplementary Table S2. GHP-88309 repeat oral dose plasma PK in ferrets. Selected plasma PK parameters of GHP-88309 in ferrets after a single oral dose of 50 or 150 mg/kg

ID	**dose**[A] [mg/kg]	t_{max} [hours]	C_{max} [nmol/ml]	**AUC-INF** [hours×nmol/ml]	**AUC-INF/dose** [hours×nmol/ml/mmol]	$t_{1/2}$ [hours]
GHP-88309	15	2	13.1 ± 0.29	49.7 ± 13.7	887.1 ± 244.1	0.84 ± 0.16
GHP-88309	50	3 ± 1	72.7 ± 17.8	359.1 ± 144.9	1913.1 ± 772.2	1.3 ± 0.66

[A]Data analysis with WinNonLin; n=3.

Supplementary Table S3. DNA primers used in this study.

species	target	sequence
Ferret	il-8_fw	5’-tgctttctgcagttctgtgtgagc-3’
Ferret	il-8_rv	5’-atgtgggccactgtcaatcactct-3’
Ferret	ifn-β_fw	5’-ggtgtatcctccaaactgctctcc-3’
Ferret	ifn-β_rv	5’-cactccacactgctgctgcttag-3’
Ferret	il6_fw	5’-agtggctgaaacacgtaacaattc-3’
Ferret	il6_rv	5’-atggccctcaggctgaact-3’
Ferret	tff1_fw	5’-ccaaggtggtctgtgttctc-3’
Ferret	tff1_rv	5’-tcctcgtcaggagagttgt-3’
Ferret	tff2_fw	5’-gagcagtgtgtgatggaagt-3’
Ferret	tff2_rv	5’-agatgaaggaaagccaggaag-3’
Ferret	tff3_fw	5’-atgcatcttctcggctgtc-3’
Ferret	tff3_rv	5’-ccactgcacattgcttcaaa-3’
Ferret	tgfbeta_fw	5’-gacatcaacgggctcagttc-3’
Ferret	tgfbeta_rv	5’-gatccacttccagcccagat-3’
Ferret	gapdh_fw	5’-aacatcatccctgcttccactggt-3’
Ferret	gapdh_rv	5’-tgttgaagtcgcaggagacaacct-3’
Ferret	ifnγ_fw	5’-tcaaagtgatgaatgatctctcacc-3’
Ferret	ifnγ_rv	5’-gccgggaaacacactgtgac-3’
Ferret	isg15_fw	5’-agcagcagatagccctgaaa-3’
Ferret	isg15_rv	5’-cagttcttcaccaccagcag-3’
Ferret	il1b_fw	5’-ttctttgaggctgatggtcc-3’
Ferret	il1b_rv	5’-acacgaaatggctcagactc-3’
Ferret	tnfa_fw	5’-ccagatggcctccaactaatca-3’
Ferret	tnfa_rv	5’- ggcttgtcacttggagttcga-3’
Ferret	muc5ac_fw	5’-gcagtccctccaagaatgaa-3’
Ferret	muc5ac_rv	5’-cacacacactggcactgata-3’
Ferret	muc5b_fw	5’-aaacgtcatcgggagtcattag-3’
Ferret	muc5b_rv	5’-atctgggtggtggagatagt-3’
CDV	cdv_n_taq_fw	5’-cgggcaagaaatggtcagaa-3’
CDV	cdv_n_taq_rev	5’-ctgagcctcttccttggtga-3’
CDV	cdv_n_probe	fam 5'-acttgccgccgagcttggca-3' bhq-1

Supplementary Figures

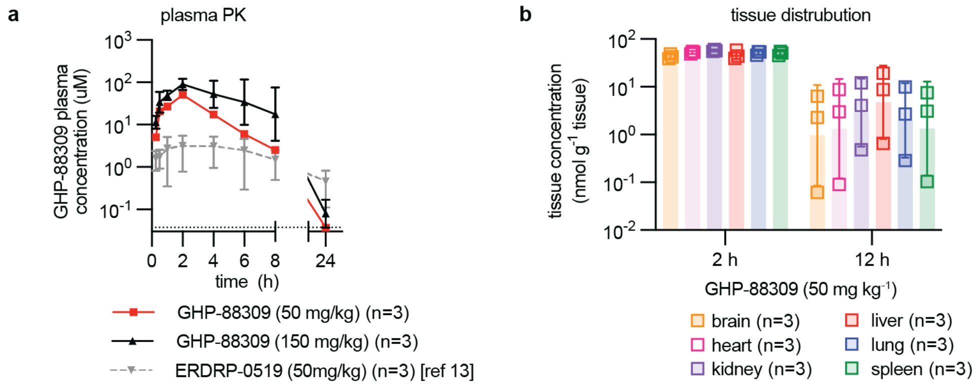

Supplementary Figure S1. Single-dose oral PK study of GHP-88309 in ferrets. a, Plasma concentration of GHP-88309 determined over a 24-hour period after a single oral dose of 50 mg/kg (red squares) or 150 mg/kg (black triangles). For comparison, historical data[1] of ERDRP-0519 plasma levels after a single oral dose of 50 mg/kg (grey triangles, dotted grey lines) are shown. **b,** GHP-88309 exposure in selected ferret organs at 2 and 12 hours after administration of GHP-88309 (50 mg/kg). Symbols represent geometric means ± geometric SD, lines intersect means (a), or independent biological repeats (b), columns denote geometric means ± geometric SD; n=3.

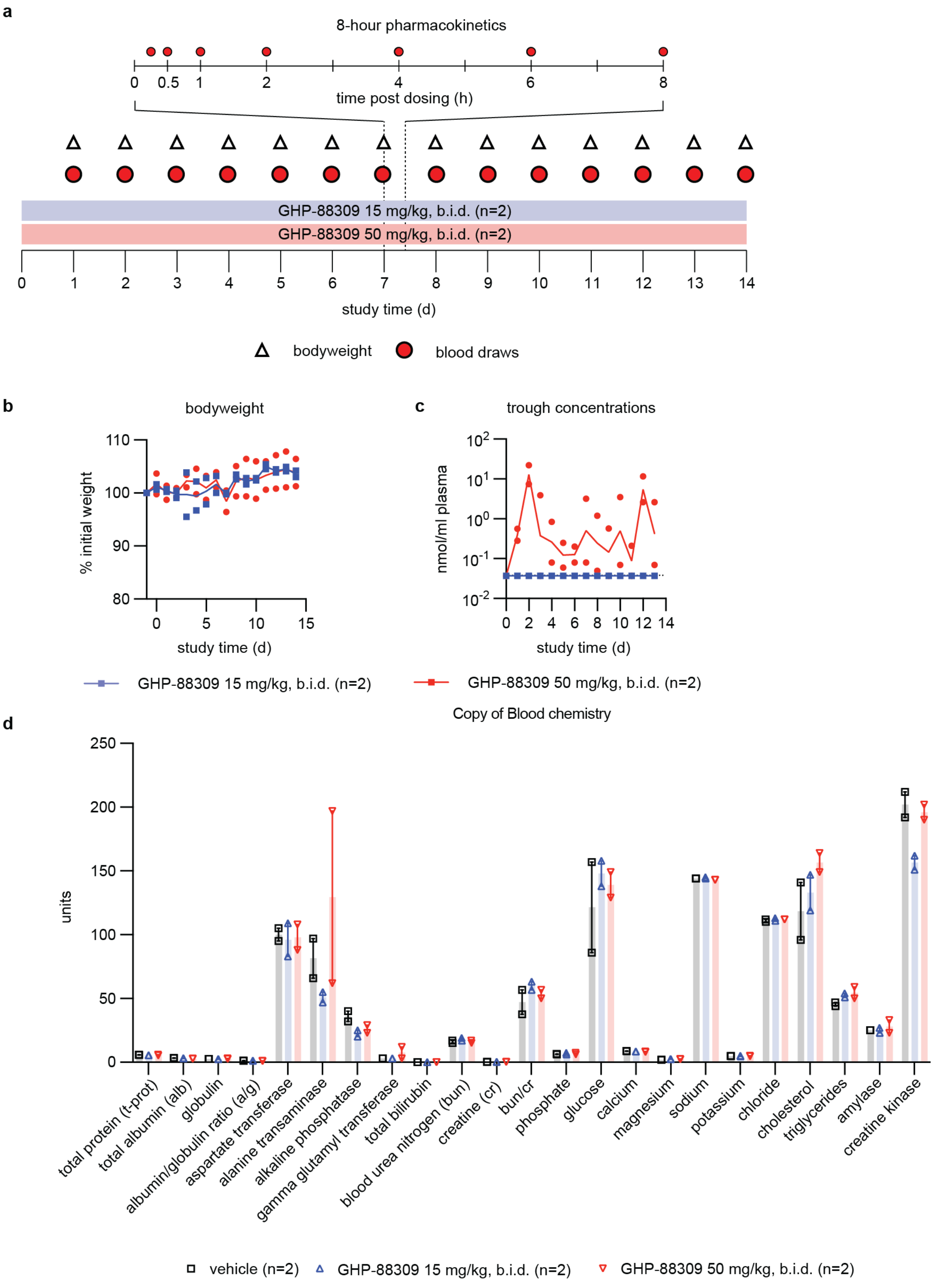

Supplementary Figure S2. Non-formal tolerability study with GHP-88309. a, Schematic of the 14-day study. Compound was delivered orally q.d. at 15 and 50 mg/kg. **b-c,** Once daily assessment of bodyweight (b) and GHP-88309 trough plasma concentrations (c). Symbols show averages of 2 animals. **d,** Select blood chemistry parameters. Symbols represent individual animals, columns show averages and range. Source data are provided as a Source Data file.

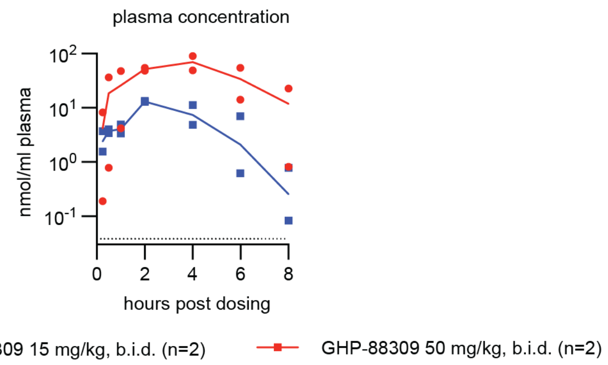

Supplementary Figure S3. GHP-88309 repeat-dose plasma PK. Ferrets were orally dosed with GHP-88309 for 7 days q.d. Shown are GHP-88309 plasma concentrations on the last day of dosing. Symbols represent individual animals, lines connect data averages. Source data are provided as a Source Data file.

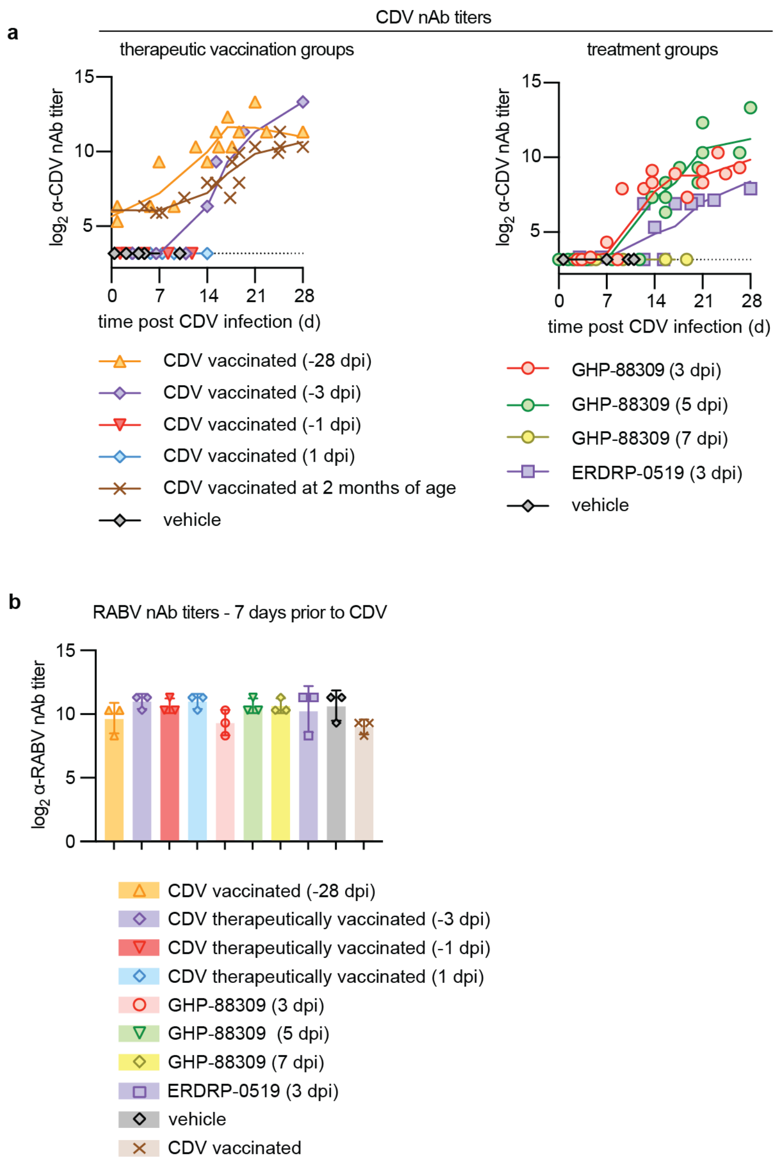

Supplementary Figure S4. α-CDV and α-RABV nAbs titers in ferrets. a-b Shown are CDV (a) and RABV (b) neutralizing titers determined using unmodified CDV-5804p or a VSV-ΔG pseudotyped with RABV G virus,

respectively. Symbols represent individual animals, lines intersect (a) and columns represent (b) geometric means ± geometric SD; n=3. Source data are provided as a Source Data file.

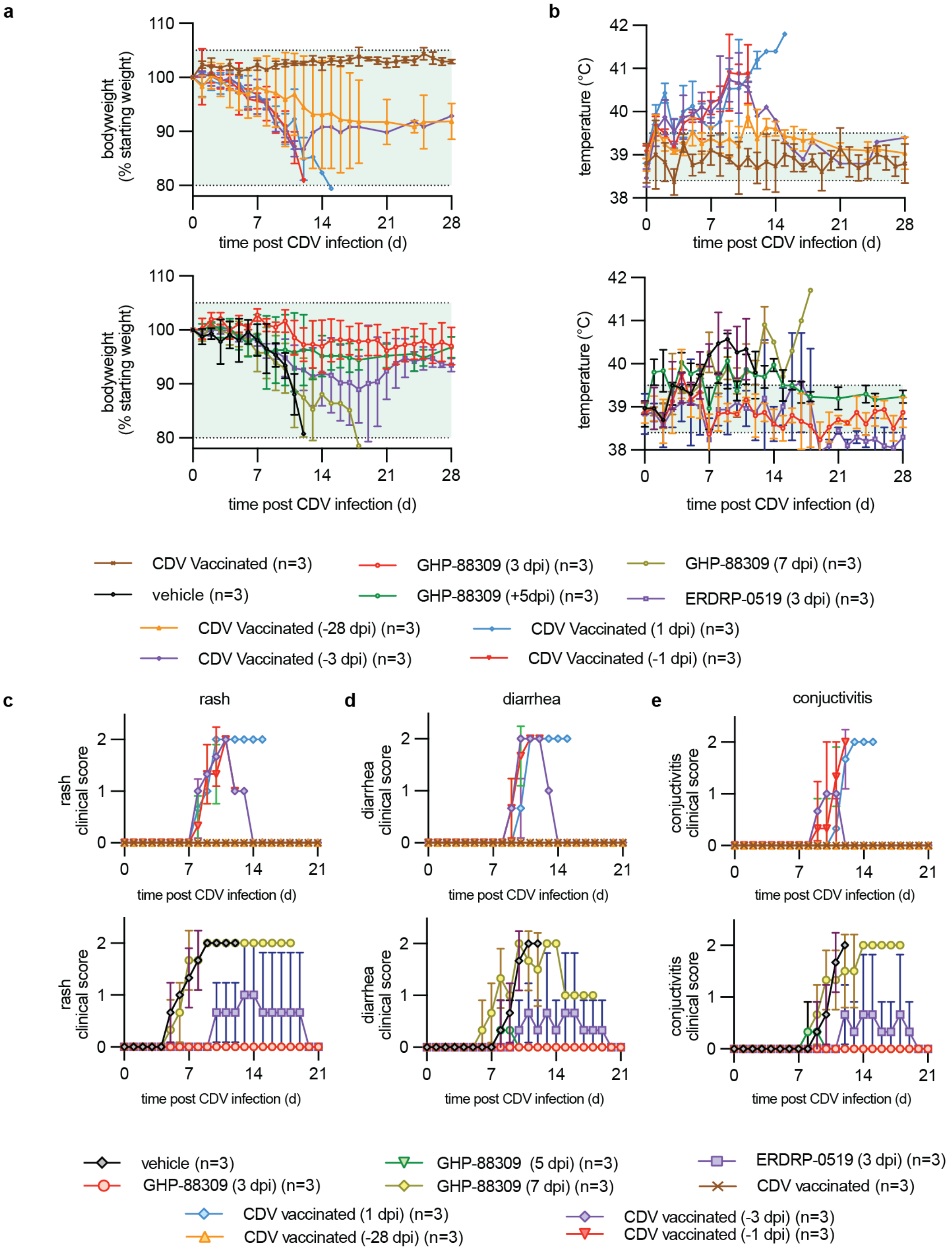

Supplementary Figure S5. Clinical signs of ferrets infected with recCDV-5804p. a-e, Bodyweight (a), temperature (b), and clinical scores of rash (c), diarrhea (d), and conjunctivitis (e) of ferrets shown in Fig. 1. Symbols represent arithmetic means ± SD, lines intersect means; n=3. Source data are provided as a Source Data file.

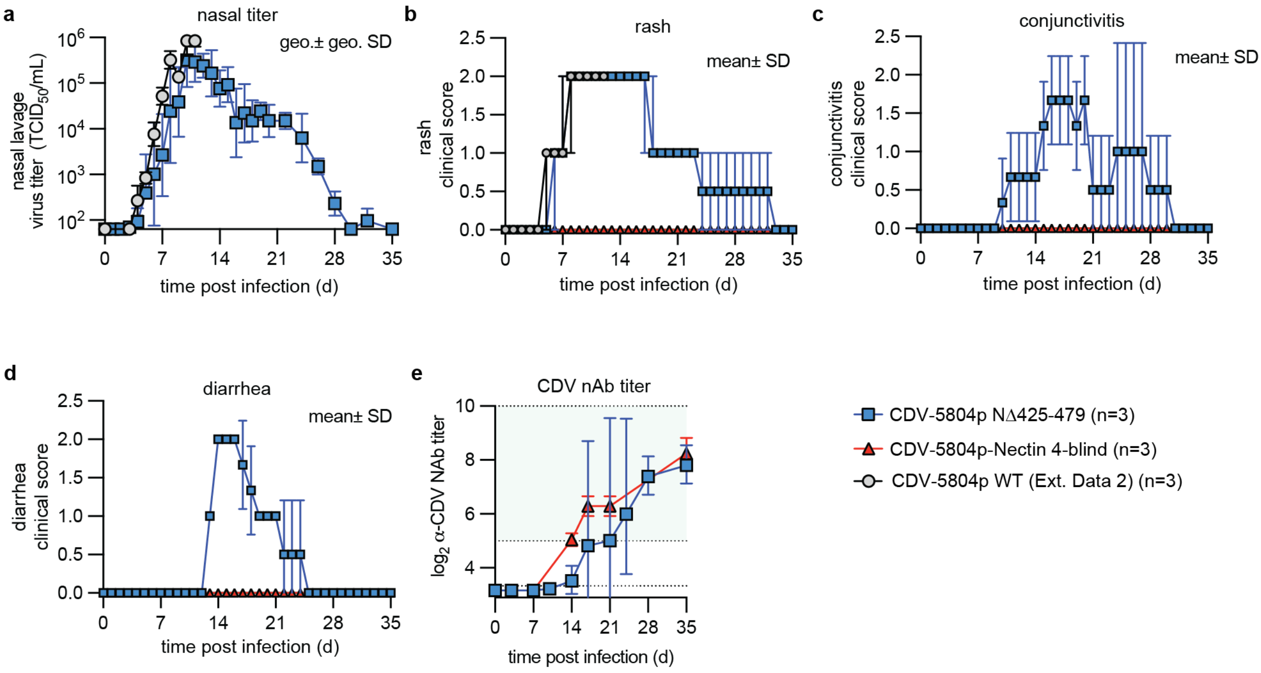

Supplementary Figure S6. Clinical disease after infection of ferrets with recCDV-5804p NΔ425-479 or recCDV-5804p Nectin 4-blind. a, Shed titers of recCDV-5804p NΔ425-479 and parental recCDV-5804p in nasal lavages obtained daily. recCDV-5804p Nectin 4-blind does not shed and is therefore not shown. **b-d,** Presentation of hallmarks of morbillivirus disease including rash (b), conjunctivitis (c), and diarrhea (d). recCDV-5804p WT is only shown in (b), since animals infected with this virus rapidly succumbed to the infection before the development of other clinical signs. **e,** Appearance of α-CDV nAbs titers in ferrets after infection with recCDV-5804p NΔ425-479 or recCDV-5804p Nectin4-blind. Shown are neutralizing titers determined using non-modified recCDV-5804p. Symbols represent geometric means ± geometric SD (a, e) or arithmetic means ± SD; green shading denotes protective nAb titers; n=3. Source data are provided as a Source Data file.

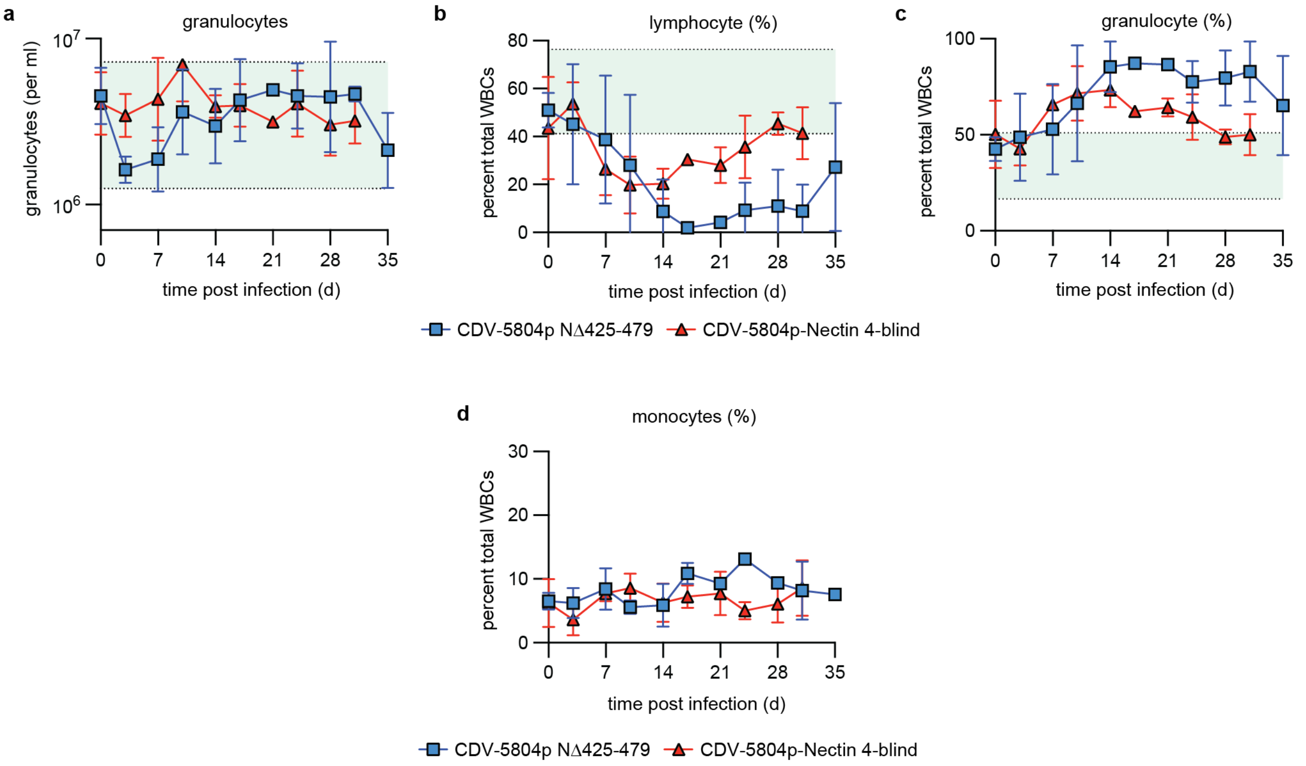

Supplementary Figure S7. CBC results after infection of ferrets with recCDV-5804p NΔ425-479 or recCDV-5804p Nectin 4-blind. a-d, Absolute quantitation of neutrophils (a) and relative quantitation of lymphocytes (b), granulocytes (c), and monocytes (d) after CDV infection. Symbols represent geometric means ± geometric SD (a, d) or arithmetic means ± SD (b, c), lines connect means. Green shadings denote normal range in uninfected animals; n=3. Source data are provided as a Source Data file.

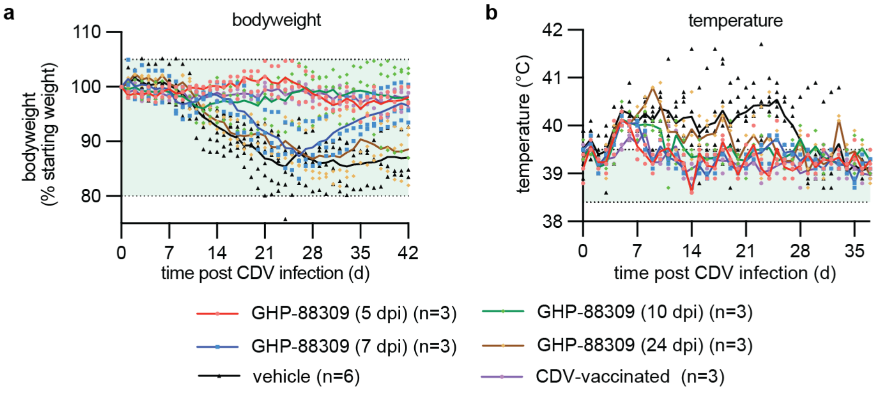

Supplementary Figure S8. Clinical presentation of recCDV-5804p NΔ425-479-infected and GHP-88309-treated ferrets. a-b, Bodyweight (a) and temperature measurements (b) assessed once daily over the course of acute CDV disease of ferrets from Fig. 3a. Source data are provided as a Source Data file.

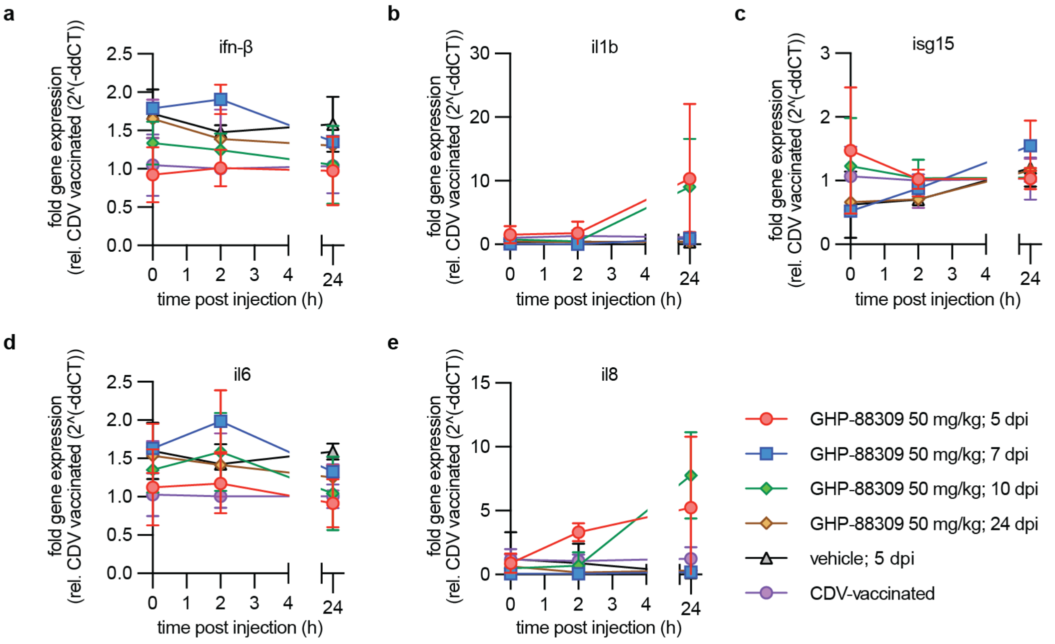

Supplementary Figure S9. Selected cytokines profiles after flagellin-stimulation of ferrets recovered from CDV. a-e, Relative changes in expression level of IFN-β (a), IL-1b (b), ISG-15 (c), IL-6 (d), and IL-8 (e) were determined in a 24-hour period after i.m. administration of purified flagellin by RT-qPCR. Symbols represent arithmetic means ± SD, lines connect means; n=3. Source data are provided as a Source Data file.

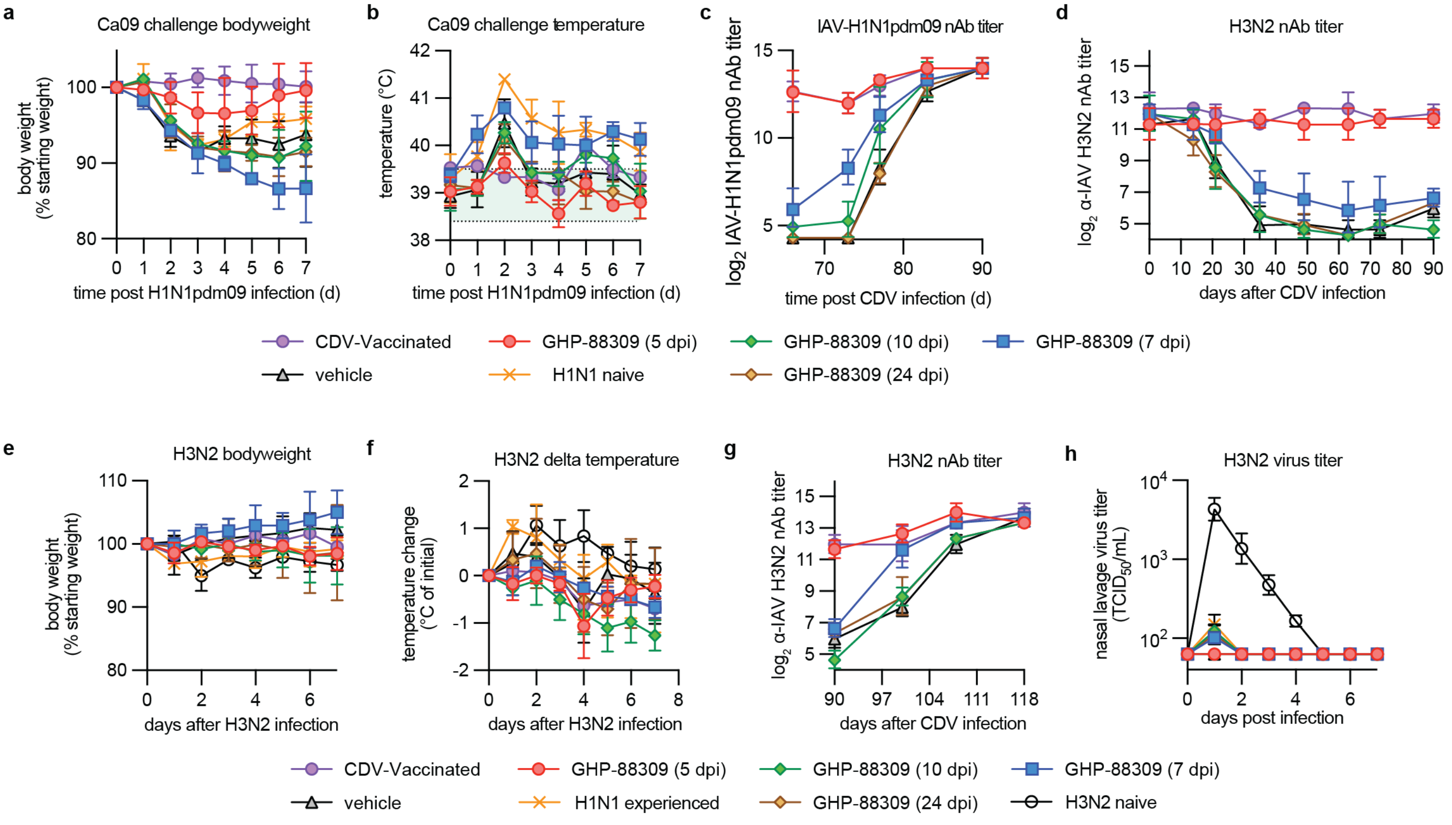

Supplementary Figure S10. IAV challenge of ferrets after recovery from CDV disease. a-c, Bodyweight (a), temperature (b), and α-IAV H1N1 nAb titers (c) of ferrets after recovery from CDV disease and infection

with pdmCA09 (H1N1) as specified in Fig. 2e; green shading in (b) denotes normal range. **d,** α-IAV H3N2 nAb titers in ferrets from Fig. 2e surviving CDV infection. **e-f,** Bodyweight and temperature of ferrets after infection with IAV-Wyo (H3N2) as specified in Fig. 2e. **g,** α-IAV H3N2 nAb titers infection with IAV-Wyo (H3N2). **h,** IAV-Wyo **(**H3N2) nasal lavage titers collected after challenge with IAV-Wyo. Symbols represent arithmetic means ± SD (a, b, d, e) or geometric means ± geometric SD (c, f, g); n=3. Source data are provided as a Source Data file.

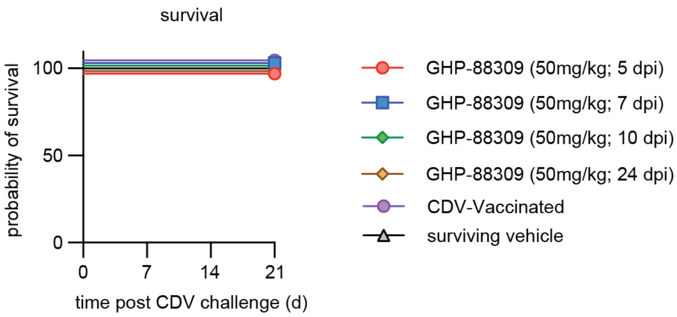

Supplementary Figure S11. Challenge of GHP-88309-treated ferrets with CDV-5804p after recovery. Animals were monitored for 21 days after challenge; n=3.

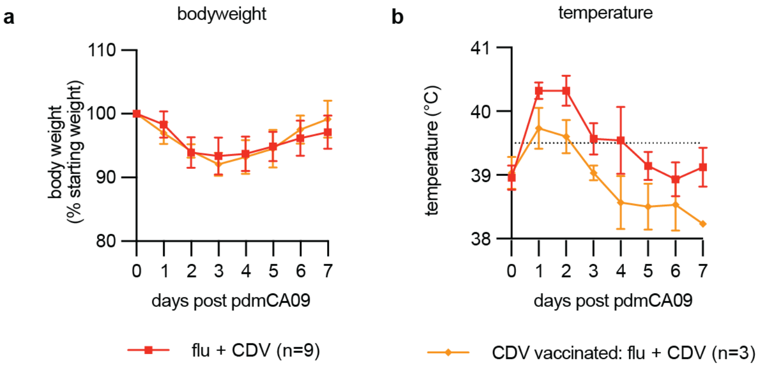

Supplementary Figure S12. Clinical disease after infection of ferrets with pdmCA09. a-b, Presentation of hallmarks of IAV disease including loss of bodyweight (a) and fever (b). Symbols represent arithmetic means ± SD, lines connect means; dotted line (b) defines fever in ferrets; n=3 or 9 as specified. Source data are provided as a Source Data file.

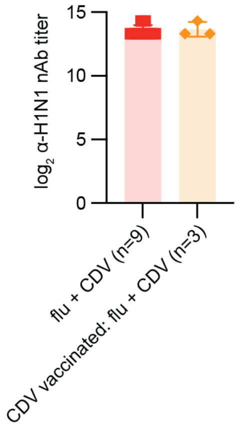

Supplementary Figure S13. α-H1N1 nAbs titers in ferrets before infection with CDV. Shown are neutralizing titers determined using pdmCA09. Symbols represent geometric means ± geometric SD; n=3 or 9 as specified. Source data are provided as a Source Data file.

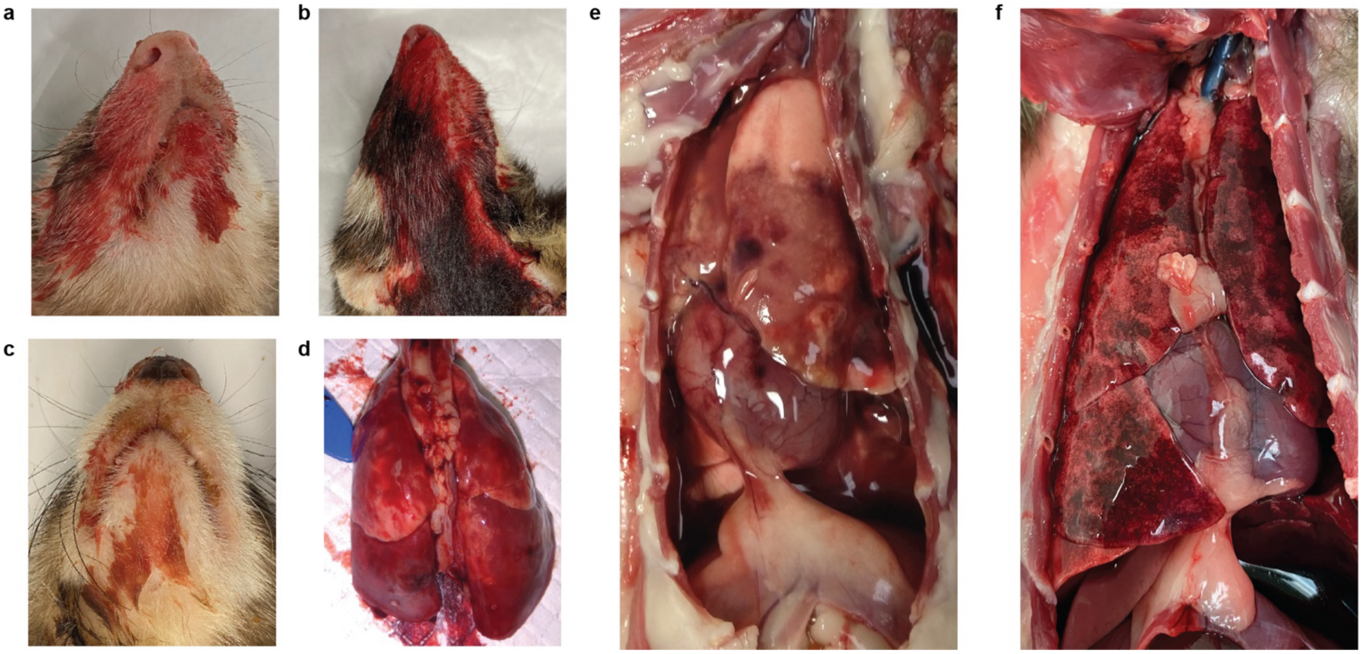

Supplementary Figure S14 Exacerbated lung disease after consecutive infection of ferrets with IAV and CDV. a-f, Macroscopic (a-c) and necropsy (d-f) presentation of hemorrhagic pneumonia in consecutively infected ferrets. Enlarged views of gross pathology of ferrets shown in Fig 4c.

flu + CDV (15 dpi; from Fig. 4)

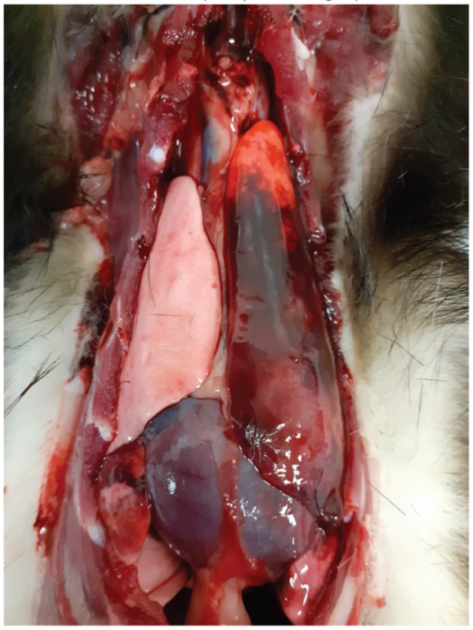

flu + CDV (15 dpi)

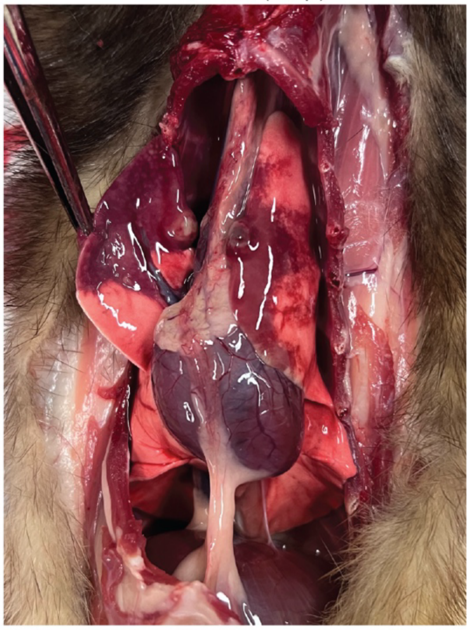

flu + CDV (16 dpi)

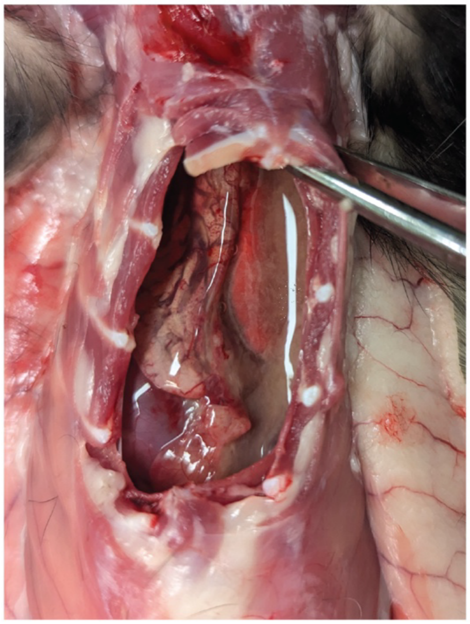

flu + CDV (18 dpi)

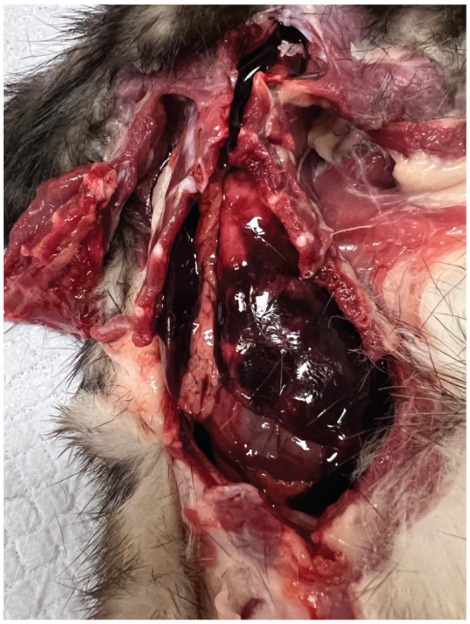

flu + CDV (16 dpi)

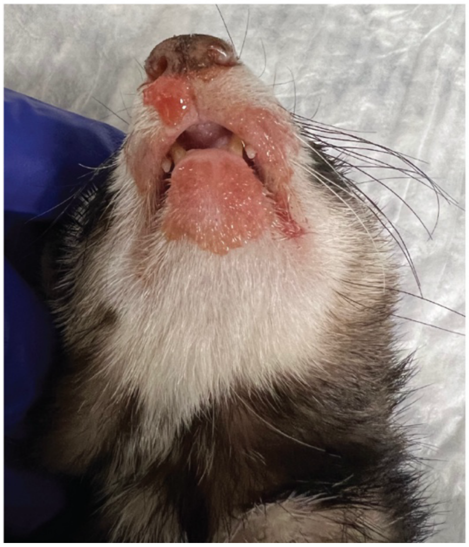

Supplementary Figure S15. Necropsy of consecutively infected ferrets presenting moribund. Shown is hemorrhagic pneumonia presentation in independent, consecutively infected animals that could be subjected to necropsy; top left, uncropped image of the animal shown in Fig. 4f.

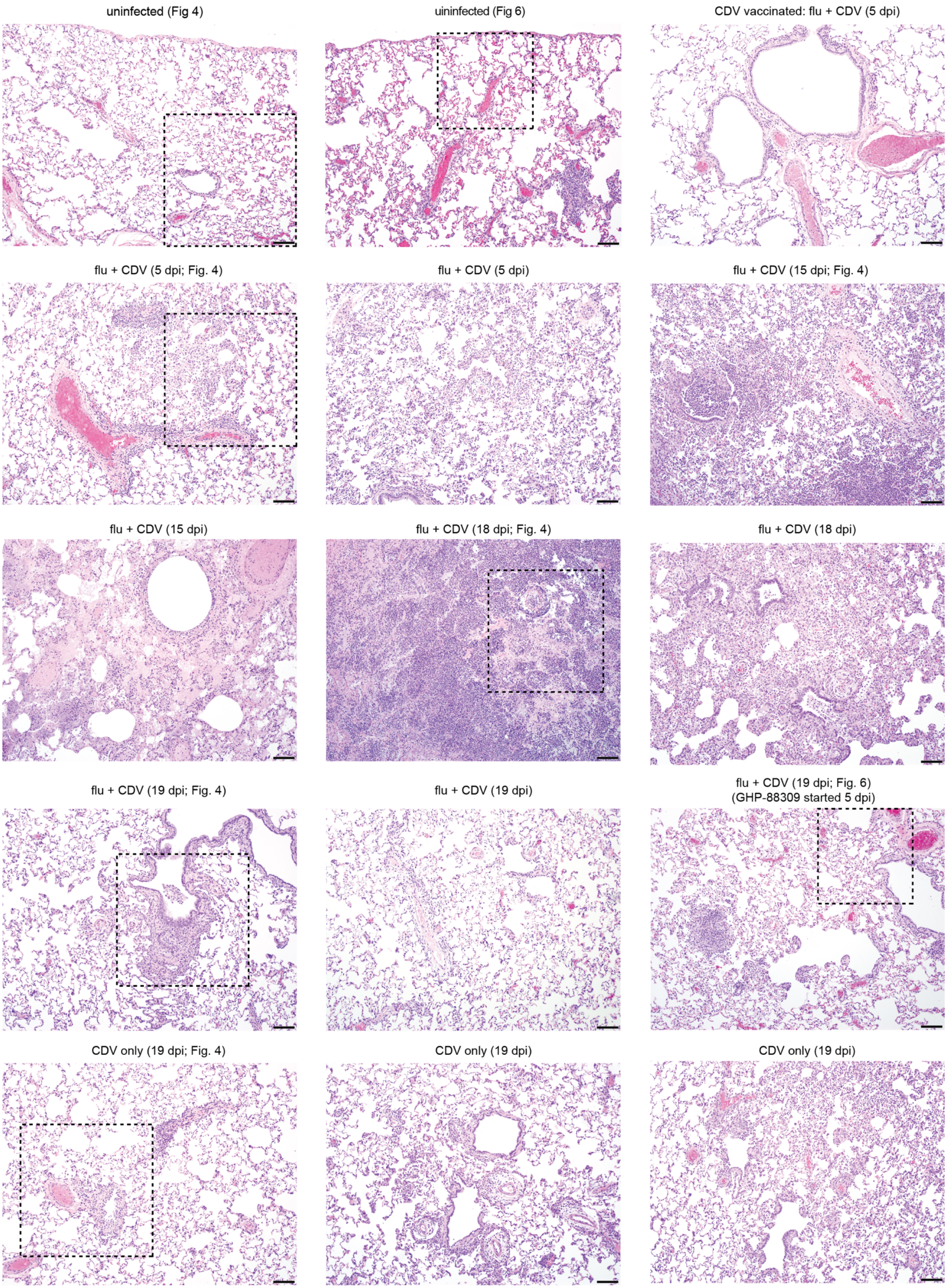
uninfected (Fig 4)
uininfected (Fig 6)
CDV vaccinated: flu + CDV (5 dpi)
flu + CDV (5 dpi; Fig. 4)
flu + CDV (5 dpi)
flu + CDV (15 dpi; Fig. 4)
flu + CDV (15 dpi)
flu + CDV (18 dpi; Fig. 4)
flu + CDV (18 dpi)
flu + CDV (19 dpi; Fig. 4)
flu + CDV (19 dpi)
flu + CDV (19 dpi; Fig. 6)
(GHP-88309 started 5 dpi)
CDV only (19 dpi; Fig. 4)
CDV only (19 dpi)
CDV only (19 dpi)

Supplementary Figure S16. All histopathology analyses. Shown are H&E-stained lung section of independent animals. Dashed squares mark the fields of view shown in Fig. 4g and Fig. 6k, respectively; scale bars represent 100 μm.

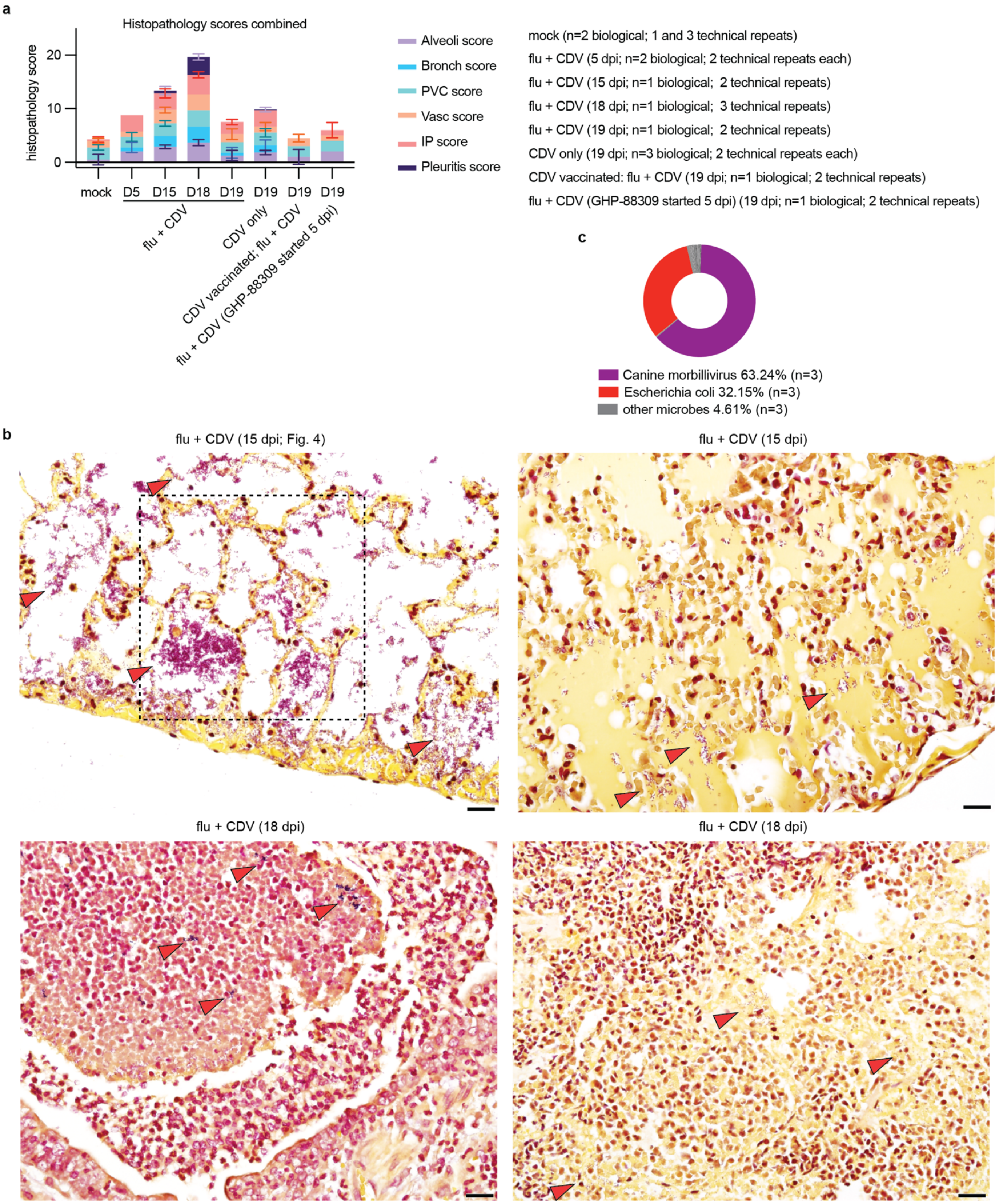

Supplementary Figure S17. Histopathology scores, Gram stains, and metagenomics after consecutive infection of ferrets. a, H&E-stained lung sections of ferrets from Fig. 4 extracted 5, 15, 18, or 19 dpi with CDV were scored as specified in Supplementary Methods. Bars represent mean scores ± SD for each criterion, n numbers as specified; for clinical scoring, technical and biological repeats were considered equally for calculation of variance. **b,** Gram stains of lung sections of independent animals. Dashed square marks the field of view shown in Fig. 4i; scale bars represent 100 μm. **c,** Metagenomics analysis of lung tissues extracted from consecutively IAV and CDV-infected animals 15, 18, or 19 dpi with CDV. Relative distribution of pathogen-specific reads is shown; n=3. Source data are provided as a Source Data file.

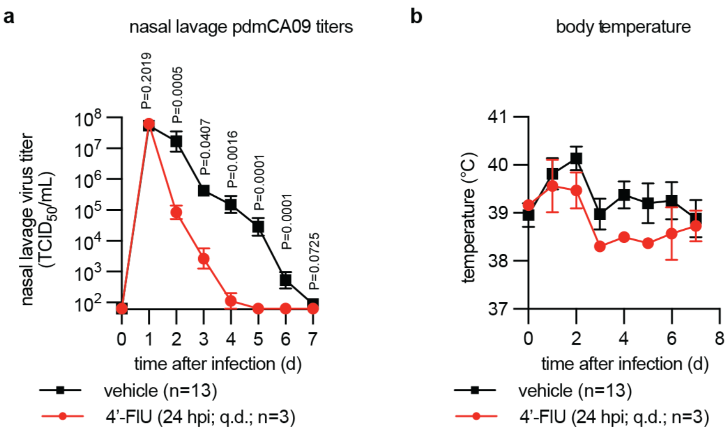

Supplementary Figure S18. Effect of treating primary H1N1-pdmCA09 infection prior to CDV. a-b, pdmCA09 (H1N1) shed virus titers (a) and body temperature (b) of ferrets infected with pdmCA09 and treated with 4'-FlU or vehicle, 28 days prior to infection with recCDV-5804p NΔ425-479 as shown in Fig. 5a. Symbols represent geometric means ± geometric SD (a) or arithmetic group means ± SD (b), lines intersect means; n numbers as specified. Source data are provided as a Source Data file.

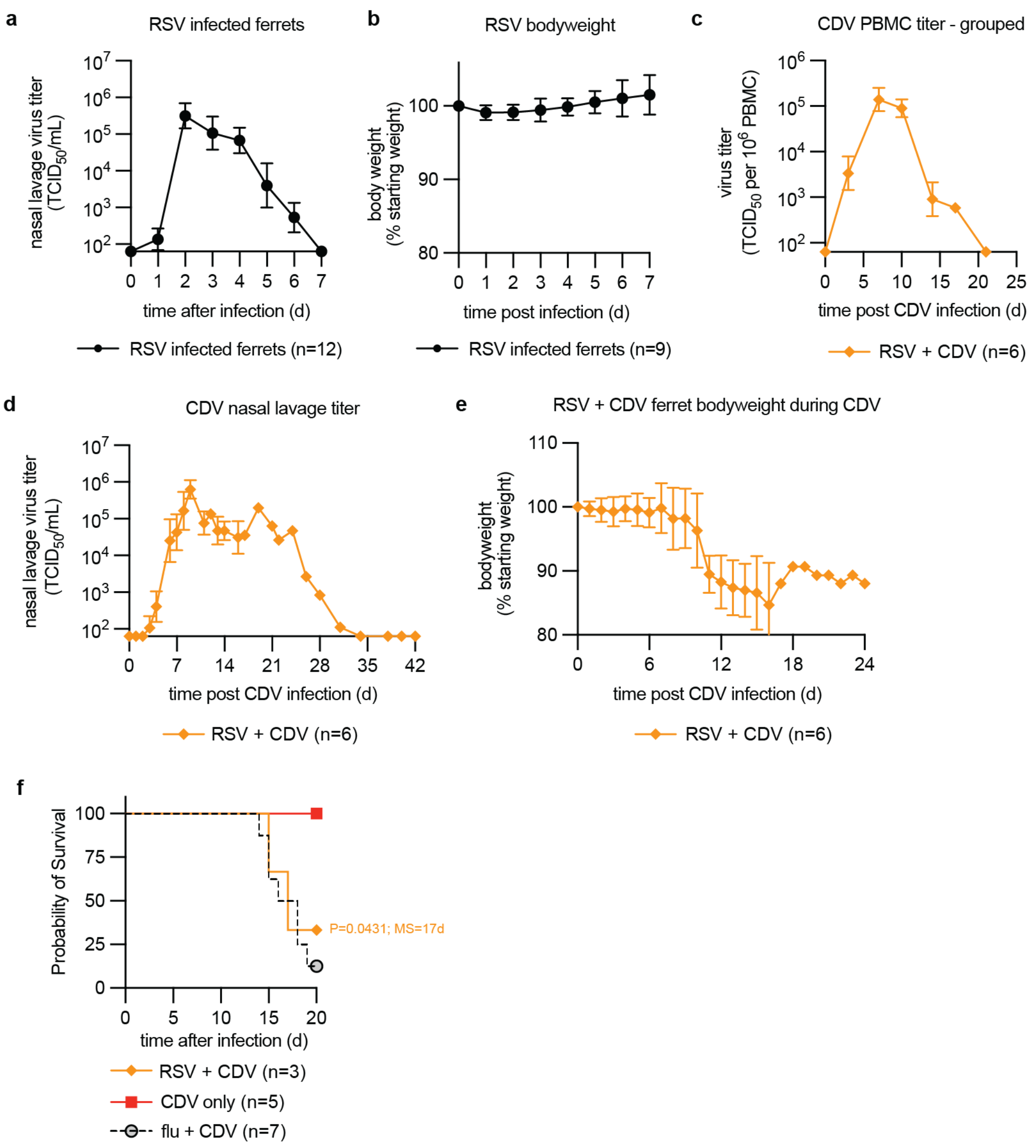

Supplementary Figure S19. Exacerbated disease after primary infection with RSV followed by recCDV-5804p NΔ425-479. a, RSV shed virus titers in nasal lavages. **b,** Bodyweight of ferrets after infection with RSV-A2-L19F. **c-f,** PBMC-associated CDV viremia (c) and nasal lavage (d) titers, animal bodyweight (e) and survival (f) after infection of ferrets with recCDV-5804p NΔ425-479 28 dpi with RSV. Symbols represent geometric means ± geometric SD (a, c, d) or arithmetic means ± SD (b, e), lines intersect means; log-rank (Mantel-Cox) test, median survival is stated (f); n numbers as specified. Source data are provided as a Source Data file.

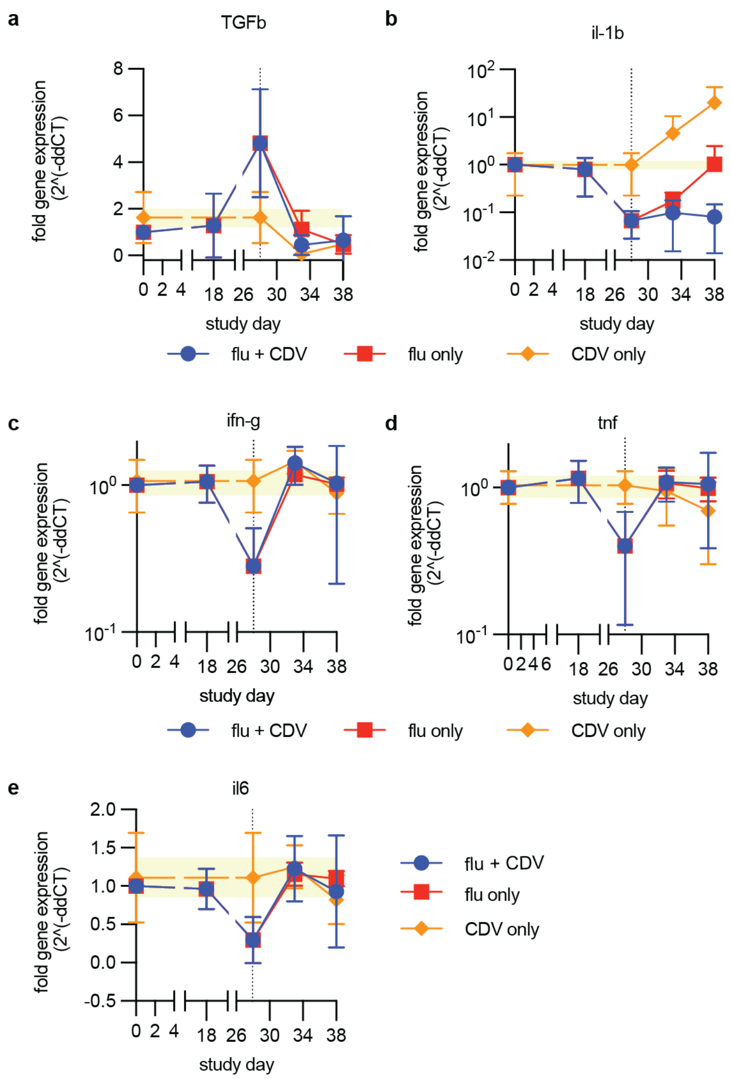

Supplementary Figure S20. Selected cytokines profiles after IAV and CDV infection of ferrets. a-e, Relative changes in expression level of TGF-β (a), IL-1b (b), IFN-γ (c), TNF (d), and IL-6 (e) message were determined at the indicated time points by RT-qPCR. Symbols represent arithmetic means ± SD, lines connect means. Dotted lines denote time of infection with CDV; yellow shading specifies variation in uninfected animals; n=3. Source data are provided as a Source Data file.

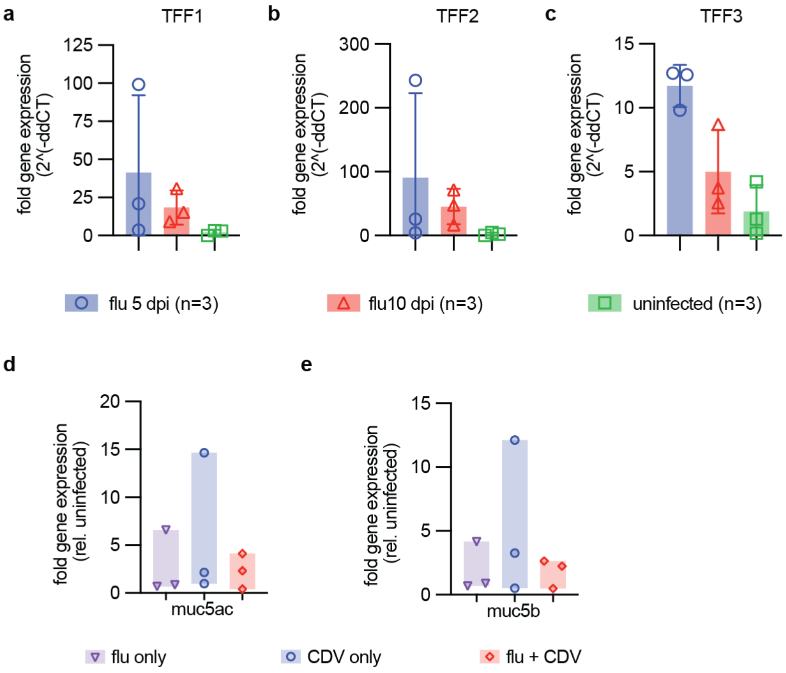

Supplementary Figure S21. Expression of TFFs and Muc5 proteins in singly or consecutively infected ferrets. a-c, RT-qPCR quantitation of relative presence of trefoil factor-encoding message in lung tissue extracted 10 dpi with pdmCA09. **d-e,** Relative changes in expression level of Muc5AC (d) and Muc5B (e) in lung tissues extracted 10 dpi with CDV were determined by RT-qPCR. Symbols represent individual animals, bars denote range from min to max; n=3. Source data are provided as a Source Data file.

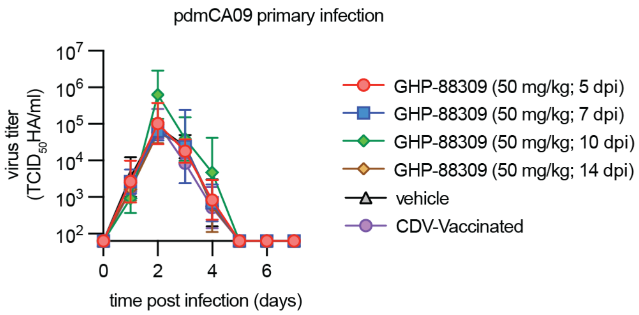

Supplementary Figure S22. Shed pdmCA09 titers after primary infection of ferrets. Shed virus titers were determined at the indicated time points through HA-$TCID_{50}$. Symbols represent geometric means ± geometric SD, lines connect means; x-axis intersects at level of detection; n=3. Source data are provided as a Source Data file.

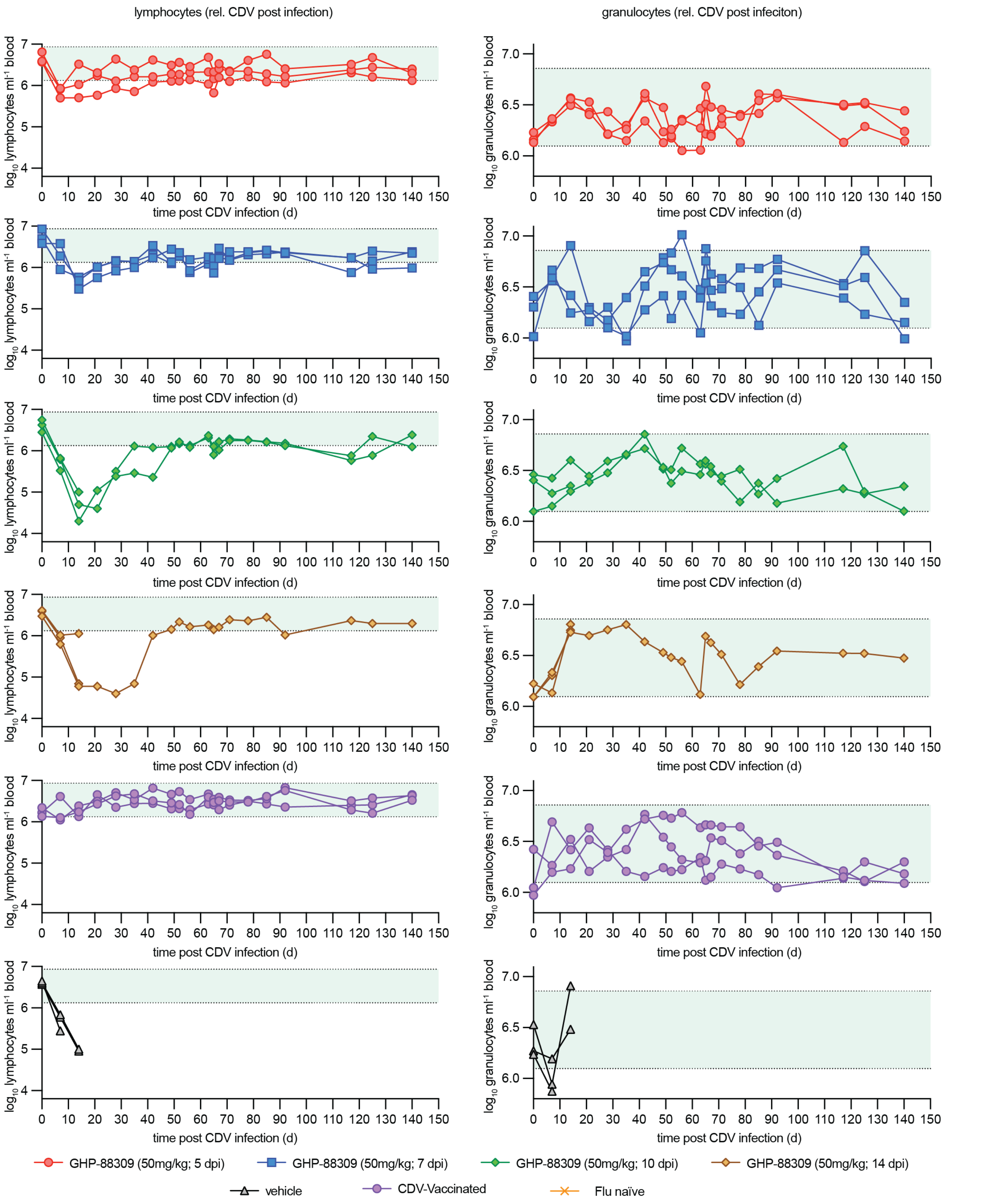

Supplementary Figure S23. CBC results of consecutively infected ferrets treated with GHP-88309.

Absolute quantitation of lymphocytes (left column) and granulocytes (right column) relative to that before CDV

infection. Symbols represent, and lines connect, individual animals. Green shadings denote normal range in uninfected animals; n=3.

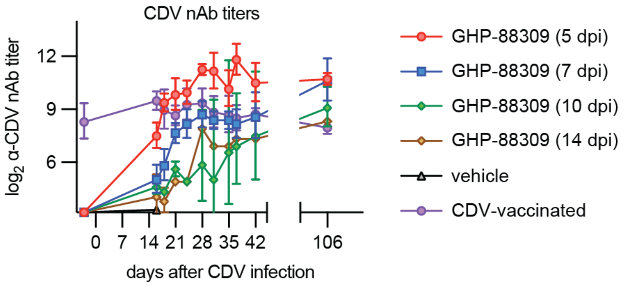

Supplementary Figure S24. α-CDV nAbs titers in GHP-88309 experienced or inexperienced ferrets after recovery from CDV. Shown are neutralizing titers determined using recCDV-5804p. Symbols represent geometric means ± geometric SD; n=3. Source data are provided as a Source Data file.

Supplementary References

1 Krumm, S. A. et al. An orally available, small-molecule polymerase inhibitor shows efficacy against a lethal morbillivirus infection in a large animal model. Sci. Transl. Med. **6**, 232ra252 (2014). https://doi.org:10.1126/scitranslmed.3008517
