# Supplementary material for: Therapeutic mitigation of measles-like immune amnesia and exacerbated disease after prior respiratory virus infections in ferrets
Source: Nat Commun. 2024 Feb 8;15:1189. doi: 10.1038/s41467-024-45418-5 (PMC10853234; doi:10.1038/s41467-024-45418-5)
Supplement: Supplementary file 3 — Description of Additional Supplementary Files [file 41467_2024_45418_MOESM3_ESM.pdf]

Description of Additional Supplementary Files

File Name: Supplementary Data 1
Description: **CDV whole genome sequencing analysis after monoinfection.** Numbers specify individual animals, D# refers to the day of sample extraction after CDV infection. 425, input virus for infection; 626.D8, 628.D8, RNA isolated 8 dpi from ferrets first treated with GHP-88309 5 dpi; 458.D8, 638.D8, 639.D8, RNA isolated 8 dpi from ferrets first treated with GHP-88309 7 dpi; 640.D9, 641.D9, 642.D9, RNA isolated 9 dpi from ferrets first treated with GHP-88309 24.

File Name: Supplementary Data 2
Description: **Metagenomics analyses.**

File Name: Supplementary Data 3
Description: **CDV whole genome sequencing analysis after consecutive infection.** Numbers specify individual animals, D# refers to the day of sample extraction after CDV infection. 425, input; 538.Dx, 539.Dx, 540.Dx, RNA isolated x dpi from ferrets first treated with GHP-88309 7 dpi; 542.Dx, 543.Dx, 544.Dx, RNA isolated x dpi from ferrets first treated with GHP-88309 10 dpi; 545.D11, 546.D11, 547.D11, RNA isolated 11 dpi from ferrets that were vehicle treated; 548.D11, 549.D11, 550.D11, RNA isolated 11 dpi from ferrets first treated with GHP-88309 14 dpi; 550.D19, RNA isolated 19 dpi from ferrets first treated with GHP-88309 14 dpi.

File Name: Supplementary Data 4
Description: **All statistical analyses.**

File Name: Supplementary Movie 1
Description: **Pulmonary ferret MRI of an uninfected animal.**

File Name: Supplementary Movie 2
Description: **Axial-slice raw data for reconstruction shown in Supplementary Movie S1.**

File Name: Supplementary Movie 3
Description: **MRI of hemorrhagic pneumonia after consecutive infection with IAV and CDV.**

File Name: Supplementary Movie 4
Description: **Axial-slice raw data for reconstruction shown in Supplementary Movie S3.**

File Name: Supplementary Movie 5
Description: **MRI 15 dpi with CDV of consecutively infected ferrets treated with GHP-88309.**

File Name: Supplementary Movie 6
Description: **Axial-slice raw data for reconstruction shown in Supplementary Movie S5.**
