# Supplementary material for: Therapeutic mitigation of measles-like immune amnesia and exacerbated disease after prior respiratory virus infections in ferrets
Source: Nat Commun. 2024 Feb 8;15:1189. doi: 10.1038/s41467-024-45418-5 (PMC10853234; doi:10.1038/s41467-024-45418-5)
Supplement: Supplementary file 14 — Reporting Summary [file 41467_2024_45418_MOESM14_ESM.pdf]

nature portfolio

Corresponding author(s): Richard K Plemper, PhD

Last updated by author(s): Jan 16, 2024

Reporting Summary

Nature Portfolio wishes to improve the reproducibility of the work that we publish. This form provides structure for consistency and transparency in reporting. For further information on Nature Portfolio policies, see our Editorial Policies and the Editorial Policy Checklist.

Statistics

For all statistical analyses, confirm that the following items are present in the figure legend, table legend, main text, or Methods section.

n/a	Confirmed	
☐	☒	The exact sample size (*n*) for each experimental group/condition, given as a discrete number and unit of measurement
☐	☒	A statement on whether measurements were taken from distinct samples or whether the same sample was measured repeatedly
☐	☒	The statistical test(s) used AND whether they are one- or two-sided<br>*Only common tests should be described solely by name; describe more complex techniques in the Methods section.*
☒	☐	A description of all covariates tested
☐	☒	A description of any assumptions or corrections, such as tests of normality and adjustment for multiple comparisons
☐	☒	A full description of the statistical parameters including central tendency (e.g. means) or other basic estimates (e.g. regression coefficient) AND variation (e.g. standard deviation) or associated estimates of uncertainty (e.g. confidence intervals)
☐	☒	For null hypothesis testing, the test statistic (e.g. *F*, *t*, *r*) with confidence intervals, effect sizes, degrees of freedom and *P* value noted<br>*Give P values as exact values whenever suitable.*
☒	☐	For Bayesian analysis, information on the choice of priors and Markov chain Monte Carlo settings
☒	☐	For hierarchical and complex designs, identification of the appropriate level for tests and full reporting of outcomes
☒	☐	Estimates of effect sizes (e.g. Cohen's *d*, Pearson's *r*), indicating how they were calculated

Our web collection on statistics for biologists contains articles on many of the points above.

Software and code

Policy information about availability of computer code

Data collection	Excel (Versions 16.52; Microsoft) and Numbers (version 10.1; Apple) used for most data collection. Reverse transcription qPCR data was collected using QuantStudio Design and Analysis (version 1.5.2; Applied Biosystems) software package.
Data analysis	Statistical analyses were performed in the Prism (GraphPad) software package (version 9.3.1). Reverse transcription RT-qPCR data were collected and analyzed using the QuantStudio Design and Analysis (version 1.5.2; Applied Biosystems) software package. Figures were composed using Adobe Illustrator (version 27.5). PK models were generated with Phoenix WinNonLin 8.3.3.33 (Certara) software package. Reverse transcription qPCR data was analyzed using the StepOnePlus software (Version 2.1; Applied Biosystems). Sequencing reads were analyzed using the TAYLOR pipeline, available at https://github.com/greninger-lab/covid_swift_pipeline (DOI 10.5281/zenodo.6142073). RNAseq data was pseudoaligned to the ferret transcriptome using Kallisto (v0.46) and RNAseq analysis was performed using R (v4.2.1). MRI was collected using the Bruker ParaVision software package (version 360.3.1). MRI image analysis was performed using ImageJ (version 2.9.0) and ITK-SNAP (verion 3.6.2-alpha). MRI model reconstructions were performed in Chimera (version 1.13.1).

For manuscripts utilizing custom algorithms or software that are central to the research but not yet described in published literature, software must be made available to editors and reviewers. We strongly encourage code deposition in a community repository (e.g. GitHub). See the Nature Portfolio guidelines for submitting code & software for further information.

Data

Policy information about availability of data

All manuscripts must include a data availability statement. This statement should provide the following information, where applicable:

- Accession codes, unique identifiers, or web links for publicly available datasets
- A description of any restrictions on data availability
- For clinical datasets or third party data, please ensure that the statement adheres to our policy

The amplicon tiling sequencing reads generated in this study have been deposited in the NCBI BioProject database under accession code PRJNA1004336 (https://www.ncbi.nlm.nih.gov/bioproject/?term=PRJNA1004336). All other data generated in this study are provided in the Supplementary Information, Supplementary Datasets S1-S4, and the Source Data file. Quantitative source data have been deposited in Figshare (https://doi.org/10.6084/m9.figshare.24076626). Source data are provided with this paper.

Research involving human participants, their data, or biological material

Policy information about studies with human participants or human data. See also policy information about sex, gender (identity/presentation), and sexual orientation and race, ethnicity and racism.

Reporting on sex and gender	This study did not involve human research participants.
Reporting on race, ethnicity, or other socially relevant groupings	N/A
Population characteristics	N/A
Recruitment	N/A
Ethics oversight	N/A

Note that full information on the approval of the study protocol must also be provided in the manuscript.

Field-specific reporting

Please select the one below that is the best fit for your research. If you are not sure, read the appropriate sections before making your selection.

[x] Life sciences [ ] Behavioural & social sciences [ ] Ecological, evolutionary & environmental sciences

For a reference copy of the document with all sections, see nature.com/documents/nr-reporting-summary-flat.pdf

Life sciences study design

All studies must disclose on these points even when the disclosure is negative.

Sample size	Appropriate sample sizes were determined using the Resource Equation and Power analysis. Unless otherwise noted, at least three samples (independent biological repeats or individual animals) were used for each group.
Data exclusions	No data was excluded from this manuscript.
Replication	All experimental data were reliably produced; the number of independent biological repeats and, when applicable, technical repeats is specified for each experiment in the figure legends. All experiments yielding quantitative raw data were independently repeated at least 3 times.
Randomization	Sourced animals were randomly assigned to cages by animal support staff blinded for the study. After a resting period of at least 72 hours, cages were randomly assigned to study groups, animals weighed, and infected as specified in figure legends.
Blinding	Animal support staff blinded for the study assigned incoming animals randomly to cages. The investigators were not blinded to group allocation for data collection and analysis for any experiment performed in this study due to size of the research group with clearance for experimentation with ferrets under ABSL2 conditions. Ferrets cannot be handled by a single investigator for the experiments reported under ABSL2 conditions and resources available did not allow involvement of additional personnel that would have been required for blinding.

Reporting for specific materials, systems and methods

We require information from authors about some types of materials, experimental systems and methods used in many studies. Here, indicate whether each material, system or method listed is relevant to your study. If you are not sure if a list item applies to your research, read the appropriate section before selecting a response.

Materials & experimental systems

n/a	Involved in the study
☒	☐ Antibodies
☐	☒ Eukaryotic cell lines
☒	☐ Palaeontology and archaeology
☐	☒ Animals and other organisms
☒	☐ Clinical data
☒	☐ Dual use research of concern
☒	☐ Plants

Methods

n/a	Involved in the study
☒	☐ ChIP-seq
☒	☐ Flow cytometry
☒	☐ MRI-based neuroimaging

Eukaryotic cell lines

Policy information about cell lines and Sex and Gender in Research

Cell line source(s)	African green monkey kidney epithelial cells (CCK-81; ATCC) stably expressing canine CD150 (Vero-cSLAM) (source Yusuke Yanagi)<br>Madin-Darby canine kidney cells (MDCK; CCL-34)<br>Human carcinoma cells (HEp-2; ATCC CCL-23)
Authentication	Cells were authenticated by the supplier or by morphological appearance and susceptibility to virus infection, including CDV.
Mycoplasma contamination	Cell lines were confirmed mycoplasma-negative when obtained from the supplier, followed by preparation and cryo-preservation of master and working stocks. Individual working stocks were replaced every three months. All cell lines in use in the laboratory were routinely retested for mycoplasma contamination in 3-months intervals and tested negative.
Commonly misidentified lines (See ICLAC register)	HEp-2 cells are indicated in the ICLAC database of commonly misidentified cell lines. Use of these cells was necessary, since they are highly permissive for respiratory syncytial virus.

Animals and other research organisms

Policy information about studies involving animals; ARRIVE guidelines recommended for reporting animal research, and Sex and Gender in Research

Laboratory animals	This study used female ferrets (Mustela putorius furo), family mustelids, genus mustela, 6-10 months of age; specified in the Abstract.
Wild animals	This study did not involve wild animals.
Reporting on sex	Biological sex of research animals was determined based on examination of external genitalia. Ferret studies were carried out with female animals only and no sex-based analyses have been performed for the following reason: per our IACUC protocol, co-housing studies may not be carried out with male ferrets, since males are very territorial and highly combative when co-housed, resulting in severe injury or death from fight wounds that would require termination of the study. Our capacity for housing large animals in ABSL-2 containment does not permit the use of singly-housed male ferrets. Overall, this study used 164 female ferrets.
Field-collected samples	This study did not involve field-collected samples.
Ethics oversight	All animal work was performed in compliance with the Guide for the Care and Use of Laboratory Animals of the National Institutes of Health and the Animal Welfare Act Code of Federal Regulations. Experiments involving ferrets were approved by the Georgia State University IACUC under protocols A22035 and A18035. All experiments using infectious CDV, RSV IAV, and VSV-deltaG were approved by the Georgia State Institutional Biosafety Committee under protocol B21029 and performed in BSL-2/ABSL-2 facilities at Georgia State University.

Note that full information on the approval of the study protocol must also be provided in the manuscript.

Plants

Seed stocks	N/A
Novel plant genotypes	N/A
Authentication	N/A
